# Developmental Gene Markers in Tumor Pathogenesis and Progression

**DOI:** 10.1155/2019/5462562

**Published:** 2019-07-08

**Authors:** Monica Cantile, Giuseppe Palmieri, Gerardo Botti

**Affiliations:** ^1^Pathology Unit, Istituto Nazionale Tumori-IRCCS-Fondazione G. Pascale, Naples, Italy; ^2^Unit of Cancer Genetics, Institute of Biomolecular Chemistry, National Research Council, Sassari, Italy; ^3^Scientific Direction, Istituto Nazionale Tumori-IRCCS-Fondazione G. Pascale, Naples, Italy

Aberrant activity of the genes that regulate cell differentiation and morphogenesis during embryonic development appears to be associated with specific oncogenic processes, from the control of cell growth, proliferation, and apoptosis to cell invasion and epithelial mesenchymal transition.

In this context, one of the most striking examples is represented by the homeobox gene family, a superfamily of transcription factors, mostly involved in the control of the identity of various regions along the body axis, from the branchial area to the tail. In particular, the deregulation of Class I Homeobox genes (HOX genes) is described as strongly associated with neoplastic transformation and disease progression in several human cancers.

The preparation of this special issue resulted in a series of 8 articles that have highlighted the deregulation of different developmental genes in tumor pathogenesis and progression. Several molecules, with different cellular functions, have been analyzed such as membrane receptors (FJX1 and SDC1), adhesion molecules (SALM3), and transcription factor (TFAP4).

Four-jointed Box 1 (FJX1) acts as a receptor for a signaling pathway that regulates growth, gene expression, and planar cell polarity. In fetal and early postnatal brain, it is expressed mainly in the primordia of layered telencephalic structures and in the superior colliculus. S. J. Chai et al. showed that the overexpression of FJX1 contributes to a more aggressive phenotype of nasopharyngeal carcinoma cells suggesting its role as a therapeutic target.

Latent transforming growth factor-beta-binding protein, LTBP2, is an integral component of elastin-containing microfibrils involved in the developing lung, pericardium, epicardium, and heart valves. Y. Huang et al. analyzed LTBP2 expression in colorectal cancer (CRC) patients suggesting that it may act as an oncogene in the development of CRC and in predicting CRC prognosis.

Synaptic adhesion-like molecule 3, SALM3, is expressed in telencephalic and diencephalic vesicles at embryonic day (E) 11.5 to E17.5. Y. Liu et al. showed that SALM3 expression in tumor cells or stroma is an independent prognostic factor in the overall survival rate of gastric cancer patients.

The transcription factor AP4, TFAP4, belonging to the basic helix-loop-helix-zipper (bHLH-ZIP) family is involved in cell maintenance in a proliferative, progenitor-like state by blocking p21 expression. T. Huang et al. described that TFAP4 is able to promote the invasion and metastasis by inducing epithelial-mesenchymal transition (EMT) and regulating MMP-9 expression via activating the PI3K/AKT signaling pathway in hepatocellular carcinoma cells.

Axis inhibitor 1, AXIN1, is involved in both positive and negative regulation of Wnt-beta-catenin signaling during embryonic development. Q. Li et al. analyzed three AXIN1 gene polymorphisms showing their implication in bladder cancer (BC) risk and suggesting new potential forecasting factors for prognosis of BC patients.

Syndecan 1, SDC1, is an integral membrane protein acting as a receptor for the extracellular matrix and involved in the maturation and release of B lymphocytes. K. Li et al. highlighted that the loss of SDC1 plays an important role in colorectal cancer malignant progression.

Finally, the contribution of DNA methylation status of several biomarkers has also been highlighted. M. Shan et al. showed a high promoter methylation frequency of homeobox genes HOXA11 and HOXD13 in breast cancer tissues.

Z. Ma et al. performed a review article in which they described the role of different methylated biomarkers including tachykinin-1 (TAC1), somatostatin (SST), and runt-related transcription factor 3 (RUNX3) in colorectal cancer patient diagnosis and monitoring.

In conclusion, this special issue collecting novel findings about the role of several development biomarkers will strengthen the hypothesis that the deregulation of genes involved in embryonic development could play a fundamental role in the pathogenic mechanisms of tumor diseases.

